# Altered cortical thickness in type 2 diabetes mellitus patients revealed by coordinate-based meta-analysis

**DOI:** 10.1186/s12902-026-02276-0

**Published:** 2026-04-16

**Authors:** Shengbo Han, Jie Li, Hongli Zhang, Liangliang Ping, Cong Zhou

**Affiliations:** 1 Jining Medical University Clinical College, Affiliated Hospital of Jining Medical University, Jining Medical University, Jining, China; 2 Department of Psychiatry, Shandong Daizhuang Hospital, Jining, China; 3 Department of Endocrinology, Affiliated Hospital of Jining Medical University, Jining Medical University, Jining, China; 4 Department of Psychiatry, Xiamen Xianyue Hospital, Xiamen, China; 5 Department of Psychiatry, School of Mental Health, Jining Medical University, Jining, China; 6 Department of Psychology, Affiliated Hospital of Jining Medical University, Jining Medical University, Jining, China; 7 Center for Evidence-Based Medicine, Jining Medical University, Jining, China

**Keywords:** Type 2 diabetes mellitus, Cortical thickness, Neurodegeneration, Seed-based d-mapping, Brain structure

## Abstract

**Background:**

Type 2 diabetes mellitus (T2DM) is associated with an increased risk of cognitive decline and neurodegenerative changes. Understanding structural brain changes in T2DM patients is crucial for developing targeted interventions.

**Methods:**

This study adheres to the PRISMA guidelines and is registered on PROSPERO (CRD420251056572). Coordinate-based meta-analysis (CBMA) was used via seed-based d-mapping (SDM) technique to analyze cortical thickness (CTh) variations between T2DM patients and healthy controls (HC). Data were retrieved from PubMed and Web of Science up to April 30, 2025, using pre-specified inclusion and exclusion criteria.

**Results:**

Significant CTh reductions were detected in the right rolandic operculum (ROL) and left superior temporal gyrus (STG) in T2DM patients. Jackknife analyses confirmed the robustness of these findings. Meta-regression suggested a negative association between BMI and CTh in these two regions, indicating that elevated BMI is associated with more pronounced cortical thinning.

**Conclusion:**

Our study highlights specific brain regions impacted by T2DM, suggesting that neurodegenerative mechanisms and clinical correlates are involved. These findings highlight the necessity for integrated metabolic and neurological strategies for T2DM management.

**Clinical trial number:**

Not applicable.

**Supplementary Information:**

The online version contains supplementary material available at 10.1186/s12902-026-02276-0.

## Introduction

Type 2 diabetes mellitus (T2DM) is a chronic metabolic disease characterized by insulin resistance and impaired glucose regulation [1]. Its pathogenesis is not yet fully understood, but genetic factors as well as environmental factors, such as being overweight and lacking physical activity, play significant roles [2, 3]. T2DM poses a significant global health challenge. As of 2021, approximately 529 million individuals worldwide were affected by diabetes, corresponding to a global age-standardized prevalence of 6.1% [4]. T2DM represents the predominant form of diabetes, accounting for 96.0% of all diabetes cases and contributing to 95.4% of diabetes-related disability-adjusted life-years (DALYs) globally [4].

T2DM can lead to complications such as neuropathy, nephropathy, and retinopathy [5, 6]. Furthermore, T2DM is closely associated with cognitive decline and an increased risk of dementia [7]. Multiple studies have suggested that in T2DM patients, the gray and white matter as well as the cerebral cortex in key cognitive-related regions, especially the temporal and parietal areas, show abnormalities. These regions are crucial for memory, information processing, and spatial cognition, and their aberrations may lead to cognitive impairment [8–10]. Poor glycemic control may be a primary contributor to these structural changes in the brain. The longer the duration of diabetes is, the more pronounced the neurodegenerative impact it may have on the brain [8]. When T2DM is diagnosed, substantial structural damage to the brain may have already occurred. Epidemiological studies have indicated that diabetes notably increases dementia risk, particularly when the duration of diabetes is under five years or hypoglycemia is present [11]. Studies have shown that T2DM is linked to neuroendocrine disorders that can progress to conditions resembling Alzheimer’s disease, characterized by insulin resistance and decreased insulin production in the brain [12]. These insulin-related deficits may lead to increased oxidative stress, neuroinflammation, vascular damage, and neuronal degeneration [13].

Magnetic resonance imaging (MRI) has emerged as a valuable tool for studying the neurological impacts of T2DM, particularly in elucidating structural brain changes. Recent advancements in structural MRI (sMRI) studies have provided valuable insights into the neuroanatomical alterations associated with T2DM, with increasing evidence highlighting cortical morphology changes as a significant finding [14]. These studies have consistently reported altered cortical thickness (CTh) in pivotal cognitive regions such as the anterior cingulate, temporal, and frontal lobes, which are closely linked to memory and cognitive functions [15]. In addition, MRI research has revealed abnormalities in subcortical structures such as the hippocampus and thalamus [14]. These structural alterations could function as early indicators of neurodegeneration and cognitive deterioration in T2DM patients, emphasizing the critical need for timely detection and intervention to reduce the disease’s neurological consequences. Nevertheless, the existing results display considerable heterogeneity, probably due to differences in sample attributes, data collection techniques, and analysis methods. Such diverse methodologies and results necessitate further large-scale, standardized studies to consolidate these observations and enhance our understanding of the neurobiological mechanisms underlying T2DM-related brain changes.

Coordinate-based meta-analysis (CBMA) serves as a robust method for detecting consistent patterns of brain structural variations across diverse neuroimaging investigations [16]. In particular, the seed-based d mapping (SDM) approach integrates peak coordinates with statistical parametric maps, thereby increasing the meta-analysis’s power and precision [17, 18]. Moreover, this approach permits various complementary analyses, such as jackknife and meta-regression, aiding in evaluating result stability and heterogeneity across studies [16]. Previous applications of SDM in other neurological and psychiatric disorders have proven its effectiveness in detecting consistent CTh changes [19–25]. Its application in T2DM research, particularly in voxel-based morphometry (VBM) studies [26, 27], has started to uncover important insights into how T2DM affects brain structure, yet the application of SDM in CTh - focused research remains underdeveloped.

The aim of the present study was to bridge these gaps by conducting a comprehensive meta-analysis using the SDM method to investigate CTh alterations in T2DM patients. We expect to identify consistent patterns of CTh changes and their potential clinical correlates, thereby deepening our understanding of the neurological impacts of T2DM and providing a foundation for future diagnostic and therapeutic strategies.

## Materials and methods

### Literature search strategy

The CBMA protocol was registered with PROSPERO (http://www.crd.york.ac.uk/PROSPERO) (registration number: CRD420251056572). This meta-analysis adhered to the Preferred Reporting Items for Systematic Reviews and Meta-Analyses (PRISMA) guidelines [28–30]. We performed systematic and comprehensive searches in the PubMed and Web of Science databases for studies published up to April 30, 2025. To ensure reproducibility, the search strategy combined Medical Subject Headings (MeSH) and free-text terms. The search keywords were (“type 2 diabetes mellitus” or “T2DM” or “type 2 diabetes” or “non-insulin dependent diabetes mellitus”) and (“cortical thickness” or “cortical thinning” or “freesurfer” or “surface-based morphometry” or “SBM”). Moreover, we manually checked the reference lists of identified studies and relevant reviews to avoid omissions.

### Study selection

The study selection process was independently performed by two authors (H.S. and Z.C.). Initially, titles and abstracts of all identified records were screened against the eligibility criteria. Subsequently, the full texts of potentially relevant articles were thoroughly assessed. Any disagreement between the two reviewers was resolved through discussion or, when necessary, by consultation with a third senior author (P.L.). This process adhered to the PRISMA guidelines, and a flow diagram illustrating the identification, screening, eligibility, and inclusion of studies is provided.

Studies were included if they met the following pre-defined criteria: (1) original research articles presenting a whole-brain analysis of CTh comparing T2DM patients with healthy controls (HC); (2) results reported as peak coordinates in Talairach or Montreal Neurological Institute (MNI) space; (3) application of a specific statistical significance threshold (uncorrected or corrected) for the group comparison; (4) published in English in a peer-reviewed journal. The exclusion criteria were: (1) meta-analyses, reviews, case reports, or conference abstracts; (2) studies that did not involve a direct voxel-wise or vertex-wise comparison between T2DM patients and HC; (3) studies from which the necessary peak coordinates or statistical parametric maps could not be retrieved, either from the publication or upon request to the corresponding authors.

### Quality assessment and data extraction

Two authors (H.S. and Z.C.) independently searched the literature, evaluated article quality, and extracted and verified data from eligible articles. Both authors independently evaluated the final studies’ quality via Müller’s neuroimaging meta - analysis guidelines [31]. We recorded the first author, cohort size, demographics (age and gender), illness duration, BMI, HbA1c%, comorbidities, and case-control difference peak coordinates for each study.

### SDM meta-analysis

We assessed regional CTh differences between T2DM patients and HC using the SDM software v5.15 (http://www.sdmproject.com) [17, 32]. The SDM technique combines effect sizes with peak coordinates extracted from databases containing statistical parametric maps. It recreates maps reflecting the original CTh effect size differences between patients and controls, rather than merely estimating the probability of a peak [16]. The SDM meta-analysis was performed following well-established procedures [33–35], and are briefly summarized below: (1) Peak coordinates of all CTh from each dataset were extracted at the level of *t*-statistics (*Z*- or *P* values for significant clusters which were then converted to *t*-statistics via the SDM online converter); (2) specific peak coordinates were recreated using a standard MNI map of the CTh group-difference effect sizes via an anisotropic Gaussian kernel [18]. A relatively wide full width at half maximum (20 mm) and CTh templates were used to control false-positive results; (3) Standard meta-analysis was performed to generate a mean map through voxel - wise random - effects mean calculation of study maps. According to the developers of the SDM [16], an uncorrected *P* = 0.005 using the AES-SDM software approximates a corrected *P* = 0.025. We applied stricter thresholds: uncorrected *P* value < 0.001, peak height threshold *Z* = 1.00 and cluster size threshold = 10 voxels. Visualization of the meta-analytic results on the cortical surface was performed using BrainNet Viewer [36] to optimally represent the topography of cortical thickness changes.

### Sensitivity, heterogeneity, and publication bias analyses

To evaluate the replicability of the results, we conducted a systematic whole-brain voxel-based jackknife sensitivity analysis. This procedure involved repeating the main statistical analysis *n* times for each result, excluding a different study each time. If a brain region remains significant across most study combinations in the jackknife analysis, the finding is deemed highly replicable [17].

We extracted peak coordinate values to assess heterogeneity and potential publication bias. Heterogeneity between studies was evaluated using the *I²* statistic, with values below 50% indicating low heterogeneity and thereby supporting the robustness of findings. Publication bias was evaluated for each significant cluster using Egger’s regression test, which assesses funnel plot asymmetry. A *P*-value < 0.05 was considered indicative of statistically significant publication bias.

### Meta-regression analysis

In alignment with prior meta-analyses and AES-SDM author recommendations [17], we adopted a stricter threshold (*P* < 0.0005) to account for the potential influences of mean age, disease duration, BMI, and HbA1c% on CTh abnormalities. Only brain regions identified in the main effect were considered.

## Results

### Included studies and sample characteristics

**Fig. 1:**
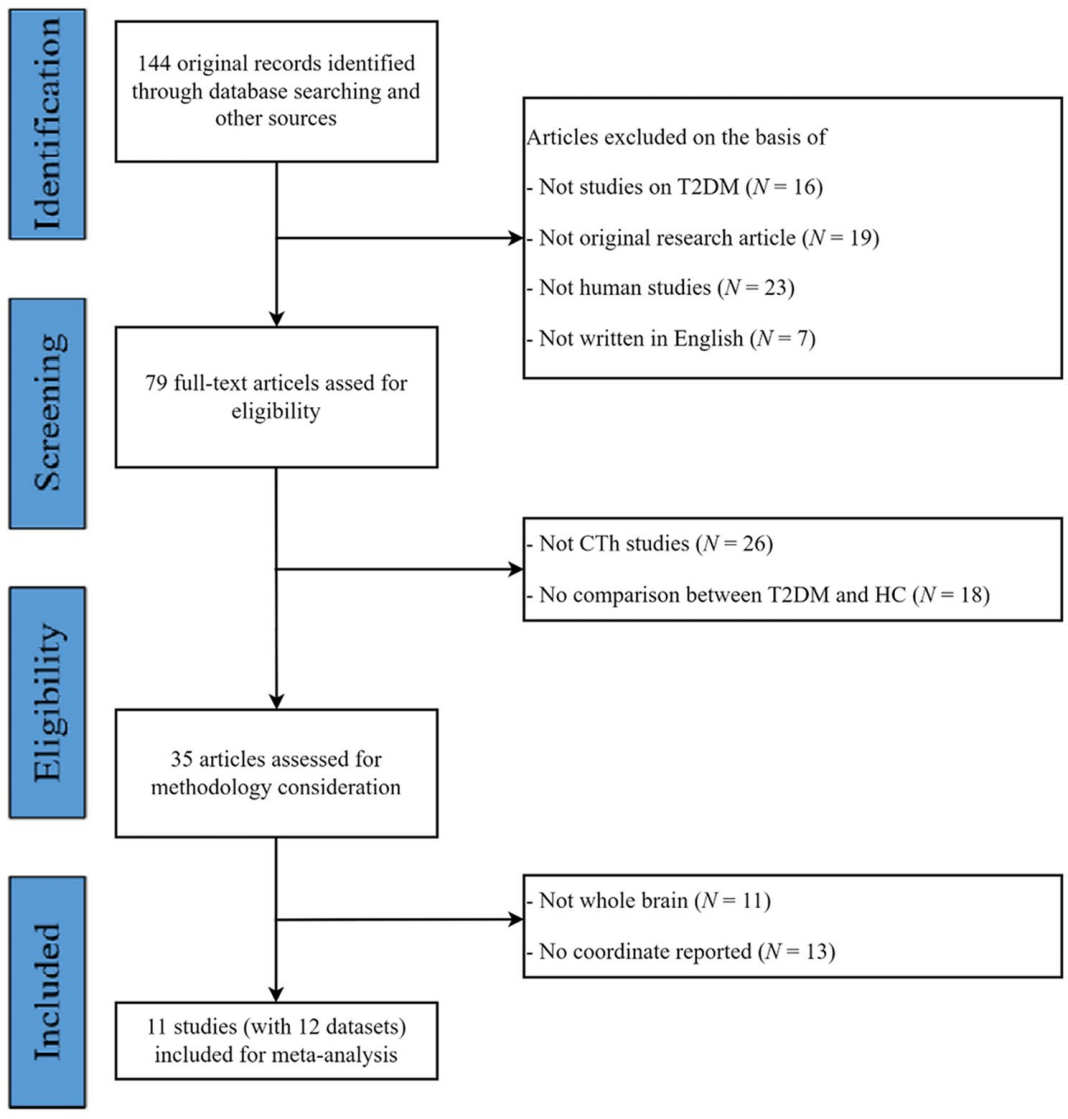
Flow diagram for the identification and exclusion of studies

The study identification and attribute flow diagram are shown in Fig. 1. The sample demographics are summarized in Table 1. Our search identified 144 studies, 11 of which met the inclusion criteria [37–47]. Among them, one study included two T2DM subgroups (painless diabetic peripheral neuropathy and painful diabetic peripheral neuropathy). Consequently, our final sample comprised 674 T2DM patients and 818 HC, with 55 coordinates extracted from 12 datasets. Some clinical variables were reported as “N/A” (Not Available) in Table 1, indicating that these data were not provided in the original publications and were unavailable from the corresponding authors upon request. A detailed summary of the neuroimaging methodologies employed in the included studies, including scanner specifications, acquisition parameters, and statistical thresholds, is provided in Supplementary Table S1.

**Table 1:** Demographic and clinical characteristics of the participants in 11 studies (12 datasets) included in the meta-analysis

Study	Subjects, *n* (female, *n*)		Age, years		Diabetes duration, years	HbA_lc_%	BMI kg/m^2^	Comorbidity (number of patients)
	T2DM	HC	T2DM	HC				
(Chen et al., 2015)	11 (8)	11 (8)	61.2	56.2	4.7	N/A	N/A	Hypertension (3)
(Shaw et al., 2017)	48 (22)	212 (116)	62.9	63.1	N/A	N/A	30.0	Hypertension (35)
(Yoon et al., 2017)	100 (50)	50 (25)	49.2	49.0	N/A	7.1	25.5	Overweight/obese (50)
(Bernardes et al., 2018)	28 (13)	31 (15)	60.4	57.1	8.2	7.0	32.3	Obesity (28)
(Liu et al., 2019)	33 (20)	24 (14)	49.1	52.5	2.3	7.3	25.4	N/A
(Zhang et al., 2019) a	44 (16)	88 (32)	54.1	55.6	7.4	9.4	23.7	Diabetic peripheral neuropathy (44) Diabetic retinopathy (13)
(Zhang et al., 2019) b	23 (12)	88 (32)	58.7	55.6	10.2	9.2	23.3	Diabetic peripheral neuropathy (23) Diabetic retinopathy (7)
(Crisóstomo et al., 2021)	86 (35)	40 (20)	60.7	57.7	12.1	9.4	29.7	N/A
(Huang et al., 2022)	78 (49)	74 (48)	47.0	47.8	4.1	9.1	24.1	N/A
(Kang et al., 2022)	44 (18)	45 (18)	50.4	49.9	4.5	8.3	24.1	N/A
(Tang et al., 2023)	29 (6)	25 (8)	41.6	37.0	4.0	9.8	22.5	N/A
(Shen et al., 2025)	150 (62)	130 (60)	50.5	51.0	5.0	9.3	24.1	Cognitive impairment (N/A)

BMI: body mass index; HbA_1c_: glycosylated hemoglobin A_1c_; HC: healthy controls; N/A: not available; T2DM: type 2 diabetes mellitus

### Pooled vertex-wise meta-analysis

**Fig. 2:**
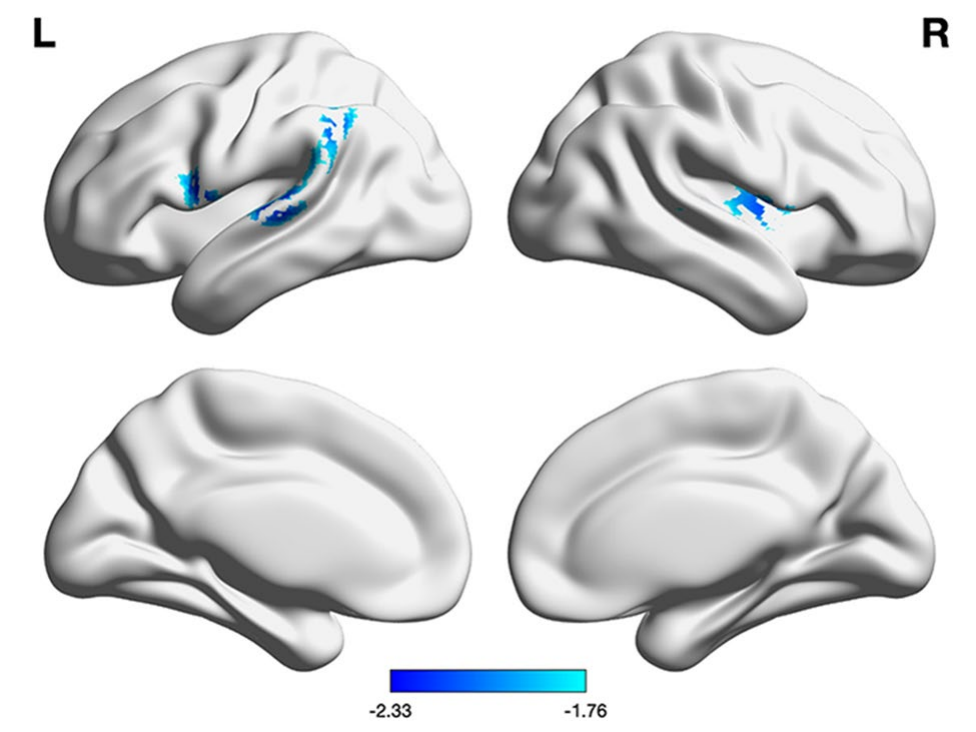
Regions showing decreased cortical thickness in type 2 diabetes mellitus patients. The results are projected onto a standard cortical surface template in BrainNet Viewer to visually represent the spatial distribution of thinning. The color bar indicates the SDM value (effect size), where negative values denote cortical thinning. For detailed Montreal Neurological Institute coordinates and a full breakdown of the cluster extents, please refer to Table 2

The pooled meta-analysis revealed two significant clusters of reduced CTh in patients with T2DM compared to HC. The primary cluster was located in the right rolandic operculum (ROL, MNI peak coordinate: x = 52, y = 0, z = 6; SDM value = -2.314), and the secondary cluster was identified in the left superior temporal gyrus (STG, MNI peak coordinate: x=-58, y=-22, z = 6; SDM value = -2.323). Both clusters were significant at a threshold of *P* < 0.001. The spatial extent and anatomical breakdown of these clusters, which included adjacent areas such as the insula and Heschl’s gyrus, are detailed in Table 2 and visualized in Fig. 2.

**Table 2:** Cortical thickness reductions in T2DM Patients compared to healthy controls

Regions	Maximum					Cluster		Jackknife sensitivity analysis
	MNI coordinates			SDM Value	*P*	Number of voxels^*^	Breakdown (number of voxels)	
	X	Y	Z					
Right rolandic operculum, BA 48	52	0	6	-2.314	~ 0	854	Right insula, BA 48 (382) Right rolandic operculum, BA 48 (219) Right heschl gyrus, BA 48 (90) Right superior temporal gyrus, BA 48 (62) Right insula (22) Right superior temporal gyrus (15) Right inferior frontal gyrus, opercular part, BA 48 (11) Right fronto-insular tract 4 (10) Right rolandic operculum (8) Right superior temporal gyrus, BA 38 (7) Right frontal aslant tract (6) Right temporal pole, superior temporal gyrus, BA 48 (4) Right temporal pole, superior temporal gyrus, BA 38 (4) Right fronto-insular tract 3 (3) Corpus callosum (3) Right heschl gyrus (2) Right inferior frontal gyrus, opercular part (1) Right temporal pole, superior temporal gyrus (1) Right superior temporal gyrus, BA 22 (1) (undefined) (3)	11/12
Left superior temporal gyrus, BA 22	-58	-22	6	-2.323	~ 0	758	Left superior temporal gyrus, BA 42 (93) Left superior temporal gyrus, BA 48 (86) Left rolandic operculum, BA 48 (75) Left inferior parietal (excluding supramarginal and angular) gyri, BA 40 (69) Left superior temporal gyrus, BA 22 (65) Left inferior frontal gyrus, opercular part, BA 6 (55) Left inferior frontal gyrus, opercular part, BA 44 (49) Left middle temporal gyrus, BA 21 (37) Left supramarginal gyrus, BA 48 (36) Left middle temporal gyrus, BA 22 (25) Left supramarginal gyrus, BA 40 (24) Left heschl gyrus, BA 48 (23) Left inferior frontal gyrus, opercular part, BA 48 (21) Left precentral gyrus, BA 6 (15) Left arcuate network, posterior segment (15) Corpus callosum (14) Left superior temporal gyrus, BA 41 (14) Left supramarginal gyrus, BA 42 (6) Left frontal aslant tract (5) Left superior temporal gyrus (5) Left inferior parietal (excluding supramarginal and angular) gyri, BA 39 (5) Left postcentral gyrus, BA 48 (4) Left rolandic operculum (4) Left supramarginal gyrus (2) Left inferior parietal (excluding supramarginal and angular) gyri (2) Left precentral gyrus (1) Left precentral gyrus, BA 48 (1) Left middle temporal gyrus (1) Left angular gyrus, BA 40 (1) Left superior temporal gyrus, BA 21 (1) (undefined), BA 40 (2) (undefined) (2)	10/12

Note: ^*^All voxels with *P* < 0.001

BA: Brodmann area; FA: fractional anisotropy; MNI, Montreal Neurological Institute; SDM, seed-based d mapping; T2DM: type 2 diabetes

### Sensitivity, heterogeneity, and publication bias results

Whole - brain jackknife sensitivity analysis indicated highly reliable results, as the right ROL reduction was consistent across all but one dataset combination. The left STG CTh reduction was replicated in 10 of the 12 datasets (Table 2).

The meta-analysis revealed that no areas with altered CTh showed a noteworthy degree of inconsistency (all *I²* < 50%) across the studies, suggesting no heterogeneity. Egger’s test revealed no significant publication bias for the identified clusters (all *P* > 0.05), and the funnel plots showed no substantial asymmetry upon visual inspection.

### Meta-regression analyses

Using a stringent threshold of *P* < 0.0005 to minimize spurious findings, meta - regression analyses revealed that CTh in the right ROL and left STG was negatively correlated with BMI. As BMI increases, CTh in these two regions decreases. See Table 3. No CTh changes related to other clinical characteristics were identified.

**Table 3:** Correlation between cortical thickness alterations and BMI in T2DM

Factor	Anatomic label	MNI coordinates			SDM Value	*P*	Number of voxels
		X	Y	Z			
BMI	Right rolandic operculum, BA 48	52	6	4	-2.080	0.000188768	34
	Left superior temporal gyrus, BA 22	-54	-32	10	-2.102	0.000137269	20

BMI: body mass index; MNI, Montreal Neurological Institute; SDM, seed-based d mapping; T2DM: type 2 diabetes

## Discussion

Our meta-analysis revealed significant reductions in CTh in the right ROL and left STG in individuals with T2DM compared with HC. These findings highlight specific brain regions affected by T2DM. Meta-regression analyses further indicated that increased BMI is associated with reduced CTh in these regions. These discoveries enhance our understanding of the neurobiological impacts of T2DM, suggesting that T2DM-related brain changes might underlie cognitive decline and mood disorders. These findings also indicate potential clinical correlates of cortical thinning, such as BMI, which could be valuable for predicting and managing neurological complications in T2DM patients. To our knowledge, this study represents the first CBMA focusing on cortical morphometry alterations in T2DM patients.

Cortical thinning in the right ROL of T2DM patients may reflect a crucial link between metabolic dysfunction and neural degeneration. As a core region of the sensorimotor integration network, the ROL coordinates somatosensory processing and motor execution by connecting to the primary sensory cortex, insula, and thalamus [48–50]. Previous studies have identified structural and functional changes in the ROL in T2DM patients [27, 51]. This finding is consistent with our CTh findings and further supports the notion that this brain region is susceptible to T2DM-related neurodegeneration. Our findings align with emerging evidence that T2DM-related hyperglycemia induces microstructural damage in sensorimotor pathways, potentially through multiple mechanisms [52, 53]. These findings suggest that both the functional and structural integrity of the right ROL may be compromised in T2DM patients, potentially contributing to cognitive decline and diabetic peripheral neuropathy. Notably, the right hemispheric lateralization of this finding may reflect its specialized role in interoceptive awareness and body schema maintenance [54]. These alterations could manifest clinically as subtle deficits in motor coordination and sensory discrimination, which might precede overt cognitive decline in T2DM patients [55, 56]. Our study highlights the right ROL as a critical region of interest for further research into the neurological complications of T2DM. Understanding the structural and functional changes in this brain area could provide valuable insights into the pathophysiology of T2DM-related cognitive impairment, and may help identify potential targets for early intervention and treatment.

The observed cortical thinning in the left STG of T2DM patients may serve as a structural marker of multi-system metabolic dysregulation affecting higher-order neural networks. As a hub for auditory processing and social cognition, the STG integrates language comprehension through its connections with Wernicke’s area and modulates emotional salience through projections to the amygdala and anterior cingulate cortex [57–59]. The atrophy of CTh in the STG was reported in previous surface-based morphometric analyses, and decreased gray matter volume was also identified by recent neuroimaging meta-analyses [15, 51]. In addition, reduced spontaneous abnormal brain activities have been identified in T2DM patients [60]. Our research indicates that decreased left STG CTh may be associated with the cognitive impairments often reported in T2DM patients, particularly in executive functioning and processing speed [7]. Structural and functional alterations in the left STG may also contribute to the increased prevalence of depression and anxiety in T2DM patients [10]. These findings highlight the left STG as a critical region for understanding the neurological complications of T2DM and suggest that it may serve as an early biomarker for cognitive decline and emotional regulation difficulties in T2DM patients.

Our study revealed consistent cortical thinning in the right ROL and left STG in T2DM patients, which aligns with prior neuroimaging evidence of regional vulnerability in sensorimotor integration and higher-order cognitive networks [60, 61]. The consistent cortical thinning observed in the right rolandic operculum and left superior temporal gyrus, evidenced by robust SDM effect sizes and high jackknife sensitivity, points to a targeted vulnerability of these regions in T2DM. The moderate to large effect sizes (SDM value < -2.3) suggest that these alterations are not merely statistically significant but may also have substantive clinical relevance, potentially underpinning the sensory integration and auditory-language processing deficits reported in this population. The spatial extent of the clusters hints at a network-level disruption beyond the peak coordinates. These structural alterations probably reflect a shared pathological pathway in which chronic hyperglycemia and insulin resistance induce microvascular damage and synaptic loss through advanced glycation end-product accumulation and oxidative stress, especially in brain regions with high metabolic demands [53, 62]. Beyond the identified structural changes, our findings of cortical thinning in the ROL and STG can be contextualized within the broader pathophysiological framework of T2DM. The patterns of regional vulnerability we observed are likely driven by a confluence of metabolic, vascular, and inflammatory mechanisms. Chronic hyperglycemia and insulin resistance, hallmarks of T2DM, are central to this process. Insulin resistance in the brain has been directly linked to functional disruptions in critical cognitive circuits, such as the Papez circuit, and to regional atrophy, suggesting a shared metabolic insult that compromises neuronal integrity [63]. From a vascular perspective, T2DM induces endothelial dysfunction and compromises cerebral blood flow, which can lead to chronic hypoperfusion and subsequent ischemia in watershed areas of the cortex [64]. Concurrently, a state of chronic low-grade inflammation is a key driver of cardiovascular-kidney-metabolic syndrome in T2DM patients [65]. This inflammatory milieu, characterized by elevated pro-inflammatory cytokines such as IL-1β, IL-6, and TNF-α, promotes oxidative stress and directly damages neurons and glial cells [66]. Furthermore, recent evidence highlights the role of amylin, a peptide co-secreted with insulin, which can form amyloid deposits in the cerebral microvasculature. These deposits contribute to neuroinflammation and cognitive decline, providing a direct link between pancreatic pathology and neurodegeneration in T2DM [67]. The cortical regions we identified, which have high metabolic demands and rich vascularization, may be particularly susceptible to this interplay of metabolic dysregulation, microvascular injury, and neuroinflammation, ultimately manifesting as measurable cortical thinning. The ROL, critical for sensorimotor integration, and the STG, which is central to auditory processing and social cognition, might be particularly susceptible to metabolic insults because of their dense vascularization and high synaptic activity [68–70]. Meta-regression analyses revealed that BMI was negatively associated with CTh in the identified regions. This implies that adiposity might exacerbate neurodegeneration in T2DM patients and obesity-related inflammation could accelerate cortical atrophy [71, 72]. These findings underscore the importance of integrating weight management into neuroprotective strategies for T2DM, as lifestyle interventions and pharmacotherapies targeting metabolic-inflammatory pathways might mitigate structural brain decline [71, 73]. These findings emphasize the need for integrated approaches that address both metabolic and neurological aspects of T2DM to improve the quality of life of patients. Future research should prioritize longitudinal designs to clarify the causal relationships between adiposity metrics and region-specific neurodegeneration, while exploring multimodal biomarkers to distinguish T2DM-specific endophenotypes from prodromal Alzheimer’s pathology.

Our study has several limitations. First, all the included studies were cross-sectional in design, which prevents us from establishing a causal relationship between T2DM and CTh changes. Second, significant variability existed across studies in terms of data acquisition parameters, MRI protocols, participant characteristics, and clinical variables. Although we applied meta-regression and subgroup analyses to investigate and adjust for this variability, it still poses a potential constraint on result interpretation. Third, the potential for publication bias must be acknowledged, as studies with positive findings are more likely to be published. We attempted to minimize this through a comprehensive search strategy, but it cannot be fully ruled out. Fourth, our study employed the AES-SDM method, which, while a well-established and widely used tool for CBMA when raw statistical maps are unavailable, relies on peak coordinates and heuristically determined thresholds rather than raw data from individual participants. Therefore, our findings should be interpreted cautiously and require confirmation in future primary studies with stricter multiple comparison corrections or through pooled individual participant data analyses. Finally, our meta-analysis focused exclusively on studies that assessed CTh. Other neuroimaging modalities such as sMRI, DTI, and fMRI were not included. This restricts our ability to comprehensively assess brain changes in T2DM patients and explore the potential interactions or complementary information among these different imaging metrics. Our findings also point to several promising avenues for future research. First, longitudinal studies are urgently needed to track the trajectory of cortical thinning in T2DM, establish its causal relationship with the disease, and identify the rate of neurodegenerative progression. Second, future work should integrate multi-modal neuroimaging data to provide a more comprehensive characterization of the interplay between cortical thinning, white matter integrity, and functional network alterations. Third, intervention studies targeting modifiable risk factors, such as optimizing glycemic control, implementing weight management programs, or using anti-inflammatory agents, are crucial to determine whether these structural brain changes are reversible. Finally, incorporating detailed cognitive and behavioral assessments alongside neuroimaging in large, well-characterized cohorts will be essential to elucidate the clinical significance of the identified cortical thinning and its role in the development of cognitive impairment and mental health comorbidities in T2DM patients.

## Conclusion

Our meta-analysis revealed significant reductions in CTh in the right ROL and left STG in individuals with T2DM compared with HC. These findings highlight specific brain regions impacted by T2DM and suggest that T2DM-related brain changes might underlie cognitive decline and mood disorders. Meta-regression analyses also revealed that increased BMI is associated with reduced cortical thickness in these regions. These findings highlight the need for integrated approaches addressing both metabolic and neurological aspects of T2DM to improve patient outcomes. Future research should prioritize longitudinal designs to explore causal relationships and multimodal biomarkers to better understand the neurological complications of T2DM.

## Supplementary Information

Below is the link to the electronic supplementary material.

Supplementary Material 1

## Data Availability

The dataset compiled for this study is available upon reasonable request to the corresponding authors.

## Abbreviations

AES-SDM: Anisotropic effect size seed-based d mapping
BA: Brodmann area
BMI: Body mass index
CBMA: Coordinate-based meta-analysis
CTh: Cortical thickness
DALYs: Disability-adjusted life-years
DPN: Diabetic peripheral neuropathy
DTI: Diffusion tensor imaging
FA: Fractional anisotropy
fMRI: Functional magnetic resonance imaging
HC: Healthy controls
MeSH: Medical subject headings
MNI: Montreal neurological institute
MRI: Magnetic resonance imaging
N/A: Not available
PRISMA: Preferred reporting items for systematic reviews and meta-analyses
PROSPERO: Prospective register of systematic reviews
ROL: Rolandic operculum
SBM: Surface-based morphometry
SDM: Seed-based d mapping
sMRI: Structural magnetic resonance imaging
STG: Superior temporal gyrus
T2DM: Type 2 diabetes mellitus
VBM: Voxel-based morphometry
