# Supplementary material for: Altered cortical thickness in type 2 diabetes mellitus patients revealed by coordinate-based meta-analysis
Source: BMC Endocr Disord. 2026 Apr 16;26:162. doi: 10.1186/s12902-026-02276-0 (PMC13214283; doi:10.1186/s12902-026-02276-0)
Supplement: Supplementary file 1 — Supplementary Material 1 [file 12902_2026_2276_MOESM1_ESM.docx]

**Table S1** Detailed summary of the neuroimaging methodologies employed in the included studies

| Study | Scanner | Analysis pipeline | Cortical parcellation atlas | Smoothing (FWHM) | Statistical threshold | Coordinate space | Number of coordinates |
| --- | --- | --- | --- | --- | --- | --- | --- |
| (Chen et al., 2015) | 3.0 T | FreeSurfer | Desikan-Killiany labeling system and Destrieux labeling system | N/A | *P* < 0.001, uncorrected | Talairach coordinate | 10 |
| (Shaw et al., 2017) | 1.5 T | FreeSurfer | N/A | 12 mm | *P* < 0.05, FDR corrected | Talairach coordinate | 6 |
| (Yoon et al., 2017) | 1.5 T | FreeSurfer | N/A | 20 mm | *P <* 0.05, using *Z* Monte-Carlo simulation with 10,000 iterations | Talairach coordinate | 9 |
| (Bernardes et al., 2018) | 3.0 T | FreeSurfer | Desikan-Killiany atlas | 10 mm | *P* < 0.05, FWE corrected | MNI coordinate | 1 |
| (Liu et al., 2019) | 1.5 T | FreeSurfer | Desikan-Killiany atlas | N/A | *P* < 0.05, FWE corrected | N/A | 0 |
| (Zhang et al., 2019) a | 3.0 T | FreeSurfer | N/A | 20 mm | *P* < 0.05, RFT corrected | Talairach coordinate | 9 |
| (Zhang et al., 2019) b | 3.0 T | FreeSurfer | N/A | 20 mm | *P* < 0.05, RFT corrected | Talairach coordinate | 5 |
| (Crisóstomo et al., 2021) | 3.0 T | CAT | N/A | 15 mm | *P* < 0.05, FWE corrected | MNI coordinate | 3 |
| (Huang et al., 2022) | 3.0 T | FreeSurfer | N/A | 15 mm | *P* < 0.001, corrected for the multicomparison of both hemispheres | N/A | 0 |
| (Kang et al., 2022) | 3.0 T | CAT | Desikan-Killiany atlas | 15 mm | *P* < 0.05, FWE corrected | MNI coordinate | 3 |
| (Tang et al., 2023) | 3.0 T | FreeSurfer | N/A | 10 mm | *P* < 0.05, TFCE corrected | Talairach coordinate | 0 |
| (Shen et al., 2025) | 3.0 T | FreeSurfer | Desikan-Killiany atlas | 15 mm | *P <* 0.05, cluster-wise correction for multiple comparisons and Monte-Carlo simulation corrected | Talairach coordinate | 9 |

CAT, Computational Anatomy Toolbox; FDR, false discovery rate; FWHM, full width at half maximum; MNI, Montreal Neurological Institute; N/A, not available; RFT, random field theory; TFCE, threshold-free cluster enhancement.
